# Development of a Lidocaine-Loaded Alginate/CMC/PEO Electrospun Nanofiber Film and Application as an Anti-Adhesion Barrier

**DOI:** 10.3390/polym12030618

**Published:** 2020-03-08

**Authors:** Seungho Baek, Heekyung Park, Youngah Park, Hyun Kang, Donghyun Lee

**Affiliations:** 1 Department of Biomedical Engineering, School of Integrative Engineering, Chung-Ang University, 221 Heukseok-Dong, Dongjak-Gu, Seoul 156-756, Korea; snb3712@gmail.com (S.B.); eldorado9228@naver.com (H.P.); heekxxxx315@gmail.com (Y.P.); 2 Department of Anesthesiology and Pain Medicine, Chung-Ang University College of Medicine and Graduate School of Medicine, Seoul 06973, Korea

**Keywords:** electrospinning, sodium alginate, carboxymethyl cellulose, anti-adhesion barrier, sustained release, crosslinking, lidocaine

## Abstract

Surgery, particularly open surgery, is known to cause tissue/organ adhesion during healing. These adhesions occur through contact between the surgical treatment site and other organ, bone, or abdominal sites. Fibrous bands can form in unnecessary contact areas and cause various complications. Consequently, film- and gel-type anti-adhesion agents have been developed. The development of sustained drug delivery systems is very important for disease treatment and prevention. In this study, the drug release behavior was controlled by crosslinking lidocaine-loaded alginate/carboxymethyl cellulose (CMC)/polyethylene oxide (PEO) nanofiber films prepared by electrospinning. Lidocaine is mainly used as an anesthetic and is known to have anti-adhesion effects. Our results show that drug release is regulated by the crosslinking degree of the lidocaine-loaded alginate/CMC/PEO film. The drug release behavior was confirmed by HPLC, and, as a result, an excellent anti-adhesion barrier was developed that can be applied to treat patients in the medical field.

## 1. Introduction

**Scheme 1:**
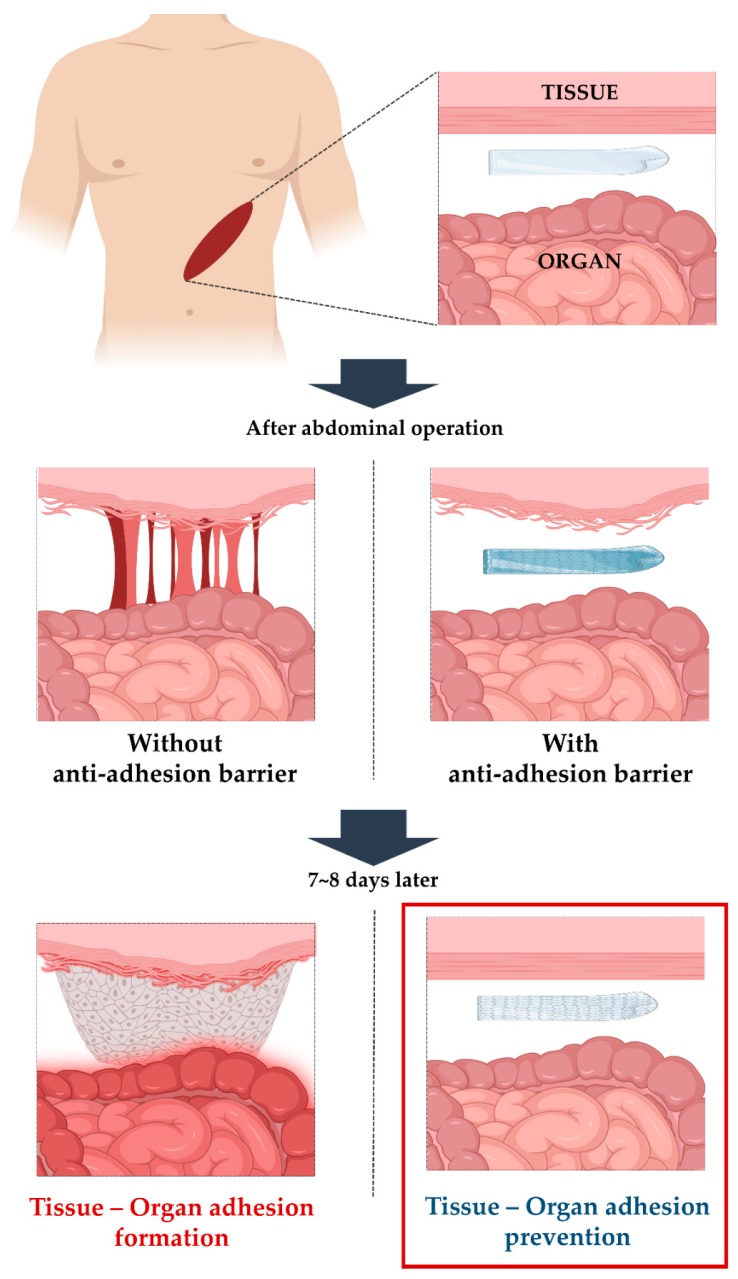
Schematic of the postsurgical tissue/organ adhesion process and the role of the anti-adhesion barrier.

Most abdominal operations cause unnecessary tissue adhesion during the tissue healing process [1], which can lead to various complications [2]. Various formulations of film or gel anti-adhesion agents are used to prevent tissue adhesion after abdominal operations [3]. The medical products normally used include Seprefilm^®^, Interceed, and Guardix-sol. Studies have demonstrated reduced tissue adhesion formation in animal models and in clinical reports [4,5,6]. Scheme 1 shows the process of postsurgical tissue/organ adhesion and the role of the anti-adhesion barrier.

As a medical product, medical sutures or medical glues are commonly used to fix the anti-adhesion agent at the surgical site [7,8]. However, suturing or glues can act as an additional stimulus to the surgical site and cause complications such as inflammation, which can potentially become another factor that causes adhesion [8,9,10]. Therefore, anti-adhesion barriers are usually applied without additional fixation in surgery.

Developing a sustained drug delivery system is important for disease prevention and treatment. Sustained drug release behavior is one way to maximize the efficacy of drugs. Excessive drug release behavior can cause side effects, and insufficient drug release behavior can make it difficult to treat diseases because of the lack of the significant effect of the drug. Therefore, many studies have been conducted to develop a system for sustained drug delivery and drug release behavior [11,12,13,14,15,16,17].

The electrospinning technique was first patented in 1934 and became the first technique for spinning nanofibers by causing electric potential differences [18]. In addition, electrospinning has been used in energy technology applications and industries. Recently, it has been widely applied in biomedical engineering and drug delivery systems (DDSs) [19,20,21,22,23,24,25,26,27]. These fields predominantly use biomaterials with various biocompatibilities and low toxicity. The mechanical properties of biomaterials can be controlled by various methods [28]. Therefore, a drug delivery carrier made by electrospinning can contain a desired quantity of drug and can control the drug release behavior through temperature, pH, and UV irradiation [23,24,26,29,30].

Crosslinking generally involves forming links between two polymer chains using crosslinking agents. These links include covalent and ionic bonds. Therefore, the control of crosslinking density is generally used to regulate the physical properties of polymers, such as drug release profiles. Numerous studies have been conducted on the regulation of drug release behavior by controlling the degree of crosslinking of drug carriers [31,32,33]. 

Biomaterials can be found in animals and plants and are extracted or obtained in various ways [34,35]. Among them, alginate is found in brown algae and can be obtained as sodium alginate [36]. Generally, alginate is known to crosslink through binding with calcium ions in aqueous solution, and has been widely applied to drug delivery systems and biomedical engineering [37,38,39,40]. In addition, alginate is a negatively charged biomaterial in aqueous solutions [41]. Therefore, alginate has low cell adhesion due to the negative charge of the cell membrane and the negative charge of the alginate surface [42]. Carboxymethyl cellulose (CMC) has been applied to tissue engineering and biotechnology by modifying cellulose in a chemical reaction. This material is also called cellulose gum, and has been studied in the form of hydrogels and nanofibers [43]. Polyethylene oxide (PEO) is a synthetic polymer that can be used to stabilize electrospinning by relieving polarization during electrospinning [44,45,46].

Lidocaine is a drug that is commonly used as a local anesthetic for mild trauma or during surgery. Various formulation studies have been conducted to maximize the efficacy of this drug and to efficiently achieve the effect of anesthesia [15,47]. Recently, studies have reported that lidocaine has not only an anesthetic effect, but also an anti-adhesion effect [48].

In this study, we used electrospinning to fabricate an alginate/CMC/PEO nanofiber film that can stably release lidocaine and developed a system that regulates drug release behavior according to the crosslinking degree. The crosslinking agent was dissolved in 95% ethanol, and the nanofiber film was prepared by finding the optimal conditions for the electrospinning solution. The lidocaine release behavior was confirmed by high-performance liquid chromatography (HPLC). Alginate is a negatively charged biomaterial with low cell adhesion. Using these properties, we designed an anti-adhesion barrier that can minimize unnecessary contact during the tissue healing process. The film developed in this study shows sustained lidocaine release behavior and excellent potential as an anti-adhesion barrier.

## 2. Experiments

### 2.1. Materials

Sodium alginate, sodium carboxymethyl cellulose and polyethylene oxide were purchased from Sigma Aldrich. Calcium chloride was purchased from GeorgiaChem (Norcross, GA, USA) for use as a crosslinking agent. For the in vitro studies, the NIH/3T3 fibroblast cell line was purchased from American Type Culture Collection (ATCC; Manassas, VA, USA). The atomic force microscopy (AFM) PPP-NCHR cantilever was selected to analyze our electrospun films and purchased from NANOSENSORS^TM^ (Neuchatel, Switzerland). To culture the cells, Dulbecco’s modified Eagle’s medium (DMEM), NewBorn Calf Serum (NBCS) and penicillin-streptomycin solution (PS) were obtained from Thermo Fisher Scientific (Waltham, MA, USA). To confirm the cell proliferation and cytotoxicity of the alginate/CMC/PEO nanofiber film, a LIVE/DEAD Viability/Cytotoxicity Kit for Mammalian Cells was purchased from Invitrogen (Carlsbad, CA, USA), and a WST-8 Proliferation Kit was purchased from Cayman Chemical (Ann Arbor, MI, USA). To evaluate cell attachment, FastWells^TM^ reagent barriers were purchased from Grace Bio-Labs^TM^ (Bend, OR, USA). A 2% lidocaine hydrochloride solution was purchased from JEIL Pharmaceutical (Seoul, Republic of Korea). In this study, this solution is referred to as lidocaine solution.

### 2.2. Preparation of the Alginate/CMC/PEO Solution for Electrospinning

To prepare a composite solution for electrospinning, sodium alginate, sodium carboxymethyl cellulose, and polyethylene oxide were mixed in a lidocaine solution with a magnetic stirrer until all the powders dissolved. Table 1 shows the three conditions considered in this study.

To produce the alginate/CMC/PEO nanofiber film without lidocaine for comparison, the composite solution was prepared under the same conditions and dissolved in water.

### 2.3. Fabrication of Electrospun Films via Electrospinning

The prepared composite solutions were transferred into a 10 mL plastic syringe with a 25 G blunt needle tip. The high-voltage connector was connected to the 25 G blunt needle tip, and the distance between the collector and the needle was set to 10 cm. The collector was made of stainless steel material, and it both contributed to the formation of potential differences for electrospinning and collected nanofibers. The voltage of the power supply was set to 25 kV to create the electric potential difference. The flow rate of the composite solution was set to 0.5 mL/h, and the electrospun film was collected on the collector.

### 2.4. Crosslinking of Electrospun Films Using a CaCl_2_ Solution

**Figure 1:**
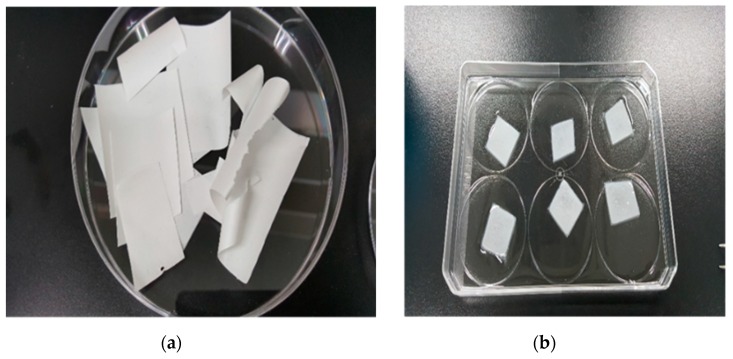
(**a**) is an alginate/carboxymethyl cellulose (CMC)/polyethylene oxide (PEO) nanofiber film obtained from the collector after electrospinning. (**b**) shows the alginate/CMC/PEO nanofiber film during the crosslinking process with CaCl_2_ solution.

Crosslinking was performed to control the drug release from the alginate/CMC/PEO nanofiber film through the control of the mechanical properties, and CaCl_2_ was selected as the crosslinking agent. For the crosslinking of the alginate/CMC/PEO nanofiber film, CaCl_2_ was completely dissolved in 95% ethanol using a stirrer. The concentrations of the crosslinking agent solutions used in this study are shown in Table 2. Before crosslinking, the electrospun film was prepared by cutting it into 1 cm × 1 cm pieces (Figure 1). Then, the cut films were placed one by one in a 6-well plate. The crosslinking reaction was carried out with the electrospun films for 30 min. Then, to remove the remaining solution, the films were washed with 95% ethanol and dried at room temperature for 30 min.

### 2.5. Cell Culture

To evaluate the cytotoxicity and proliferation of electrospun films, cell culturing was performed. NIH/3T3 cells were cultured at 37 °C and at 5% CO_2_. The cell culture basal medium was prepared with DMEM, 10% NBCS, and 1% PS. The cell medium was replaced every 2–3 days, and subculturing was carried out at 70%–80% cell confluency.

### 2.6. Evaluation of Cell Experiments on Alginate/CMC/PEO Nanofiber Films

**Scheme 2:**
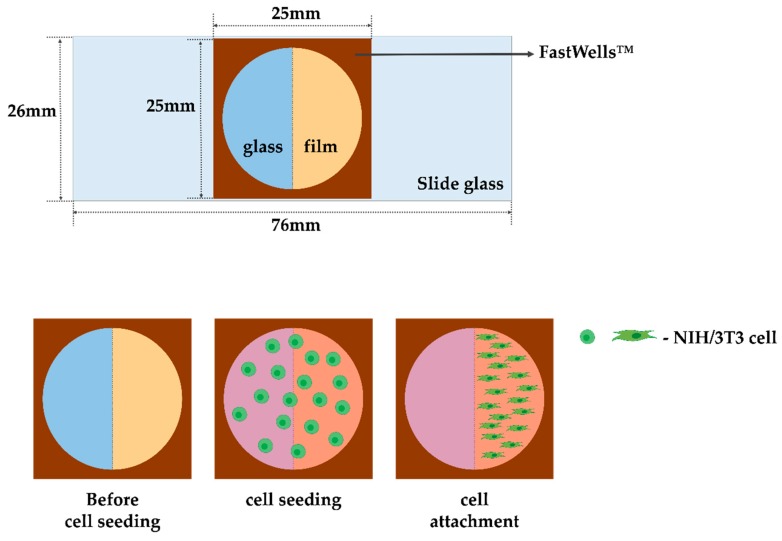
In vitro anti-adhesion test implemented in this study.

The cytotoxicity and proliferation of the electrospun films were evaluated using a LIVE/DEAD Viability/Cytotoxicity Kit for Mammalian Cells and a WST-8 Kit. To confirm the cytotoxicity of the films, NIH/3T3 cells were cultured on slide glass and placed in FastWells^TM^ reagent barriers with an electrospun film (Scheme 2). The culture environment was designed to evaluate the cell adhesion and cytotoxicity of the electrospun film. NIH/3T3 cells were seeded at 3 × 10^5^ cell density with FastWells^TM^ reagent barriers. Cytotoxicity was qualitatively evaluated by fluorescence analysis using a confocal microscope (K1-Fluo; Nanoscope Systems, Daejeon, South Korea). The proliferation evaluation of the films was performed in 96-well plates. NIH/3T3 cells were seeded at 2 × 10^5^ cell density per well. The sample was cultivated in a cell incubator for one day, and then the electrospun film was cut into 0.5 cm × 0.5 cm pieces and put into each well. After 6 h, the cell medium was removed, and 150 µL of fresh basal medium and 50 µL of WST-8 reagent were added and reacted for one day. Data on the proliferation evaluation were obtained from an absorbance analysis on the microplate reader (Synergy H1; BioTek, Winooski, VT, USA). The absorbance measurement value was performed at optical density (OD) 450 nm. 

### 2.7. Characterization of the Alginate/CMC/PEO Nanofiber Films

In this study, all the electrospun films were characterized by scanning electron microscopy (SEM; SU50000; Hitachi, Tokyo, Japan) and AFM NX10 (Park Systems, Suwon, South Korea). Before SEM operation, the electrospun films were coated with platinum by an ion sputter machine (Ion sputter, E-1010; Hitachi) for 10 min. The morphology of the electrospun films was characterized by SEM with an operating condition of 15 kV. The AFM operation conditions were performed using the PPP-NCHR cantilever in noncontact mode. To remove noise, the scan rate and drive value were set to 0.5 Hz and 20%, respectively. To analyze the chemical interactions of the electrospun films, Fourier transform infrared spectroscopy (FT-IR; Nicolet 6700; Thermo Fisher Scientific) was used, with 12 scans per sample. Before the FT-IR analysis, all samples were ground in a mortar and analyzed in powder form.

### 2.8. Preparation of HPLC Samples and Lidocaine Release Test

The lidocaine release test was performed in 6-well plates. Electrospun films were prepared at a size of 1 cm × 1 cm, and the release behavior was confirmed in a phosphate buffered saline (PBS) solution at pH 7.0. Table 3 shows the composition of the electrospun films that considered the lidocaine release behavior. The lidocaine standard sample was prepared at 1000 ppm and compared with the experimental group.

### 2.9. Statistical Analysis

All statistical analyses in this study were performed using GraphPad Prism 8 software (GraphPad Software, Inc., San Diego, CA, USA). All experimental values were derived by analyzing more than three replicates. The diameter analysis of the nanofibers was analyzed using ImageJ software.

## 3. Results and Discussion

### 3.1. Characterization of Alginate/CMC/PEO Nanofiber Films

**Figure 2:**
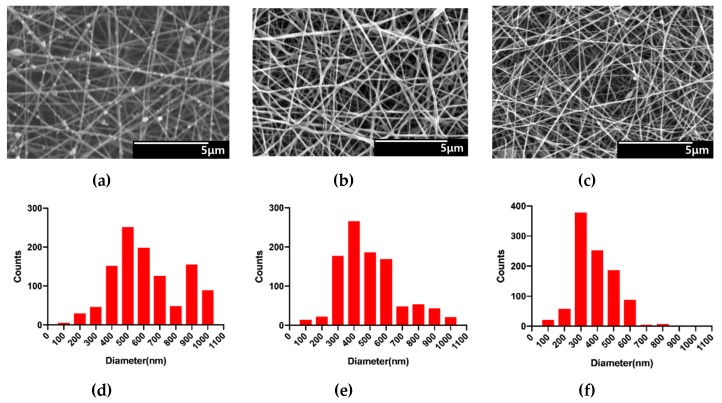
Scanning electron microscopy data and distribution plots for nanofiber-diameter alginate/CMC/PEO nanofiber films. (**a**,**d**) are data obtained from 1% (*w/v*) alginate/CMC/PEO nanofiber film. (**b**,**e**) are data obtained from 7% (*w/v*) alginate/CMC/PEO nanofiber film. (**c**,**f**) are data obtained from 9% (*w/v*) alginate/CMC/PEO nanofiber film.

**Figure 3:**
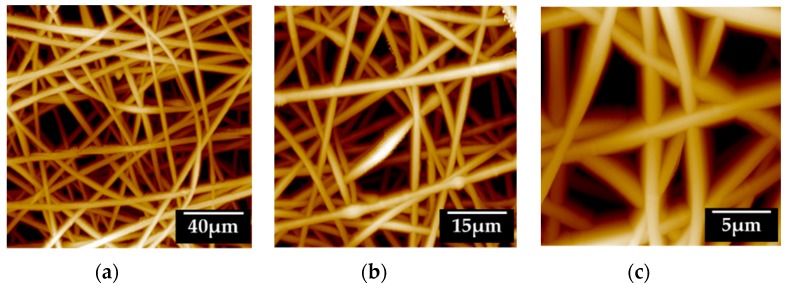
Morphological and surface characterization of the 9% (*w/v*) alginate/CMC/PEO nanofiber film. Analyses used the noncontact mode of atomic microscopy. (**a**–**c**) are the same films at different scales (scale bars 40 µm, 15 µm, and 5 µm).

The alginate/CMC/PEO nanofiber films fabricated by electrospinning were characterized by SEM. The electrospun films made under the conditions in Table 1 were analyzed, and the nanofibers were consistent under all conditions. Figure 2a,d show the 5% (*w/v*) alginate/CMC/PEO nanofiber films, and, although the nanofibers were relatively uniform, several beads were observed. Figure 2b,e show the 7% alginate/CMC/PEO nanofiber films, and the beads disappeared more severely than on the 5% alginate/CMC/PEO nanofiber film. The graph distribution shows that the nanofibers become uniform as the concentration increases (Figure 2d–f). As a result, it was confirmed that the 9% alginate/CMC/PEO nanofiber film was well made without beads, as shown in Figure 2c. In addition, atomic force microscopy clearly characterized the nanofibers of the 9% alginate/CMC/PEO film, and images were obtained using a noncontact mode. The bead form was not observed from the 9% alginate/CMC/PEO nanofiber film by atomic force microscopy (Figure 3). The 9% alginate/CMC/PEO film was uniformly distributed and well prepared without beads. Therefore, the 9% alginate/CMC/PEO film was selected as the best condition in this study.

### 3.2. Analysis of the Alginate/CMC/PEO Nanofiber Film Using FT-IR

**Figure 4:**
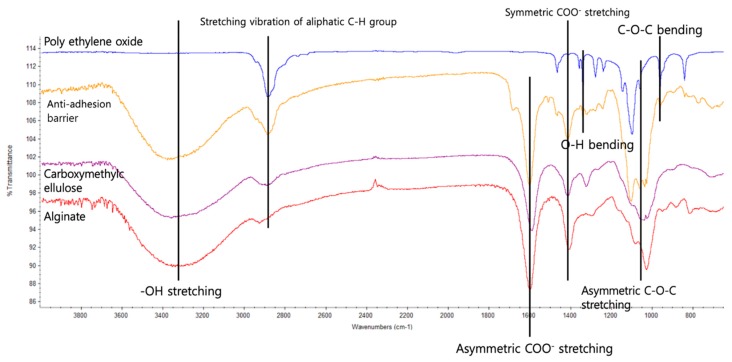
FT-IR analysis data on the alginate/CMC/PEO film (anti-adhesion barrier).

The performance of the alginate/CMC/PEO nanofiber film as an anti-adhesion barrier was measured by FT-IR. Figure 4 shows the spectrum of the electrospun film. The symmetric COO– stretching, asymmetric COO stretching and –OH stretching peaks are shown at 1407, 1601, and 3240 cm^−1^, respectively. Their peaks were observed in alginate and carboxymethyl cellulose. The peaks at 1098 and 843 cm^−1^ indicate asymmetric C–O–C stretching and C–O–C bending as PEO peaks. The O–H bending peak and stretching vibration of the aliphatic C–H group are shown at 1419 and 3280 cm^−1^, respectively. These peaks also represented PEO peaks (Figure 4). A comparison with the alginate/CMC/PEO nanofiber film (anti-adhesion barrier) prepared by electrospinning shows new peaks, and the characteristics of alginate, carboxymethyl cellulose, and PEO did not disappear, indicating that the alginate/CMC/PEO film was formed.

### 3.3. In Vitro Experiment with Alginate/CMC/PEO Nanofiber Films

**Figure 5:**
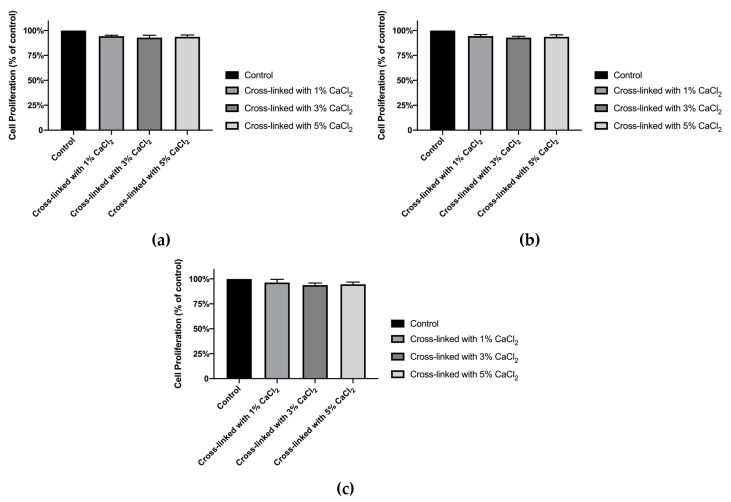
The cytotoxicity test was performed using a microplate reader (optical density (OD): 450 nm). (**a**) graph obtained from 5% (*w/v*) alginate/CMC/PEO nanofiber film. (**b**) graph obtained from 7% (*w/v*) alginate/CMC/PEO nanofiber film. (**c**) graph obtained from 9% (*w/v*) alginate/CMC/PEO nanofiber film.

**Figure 6:**
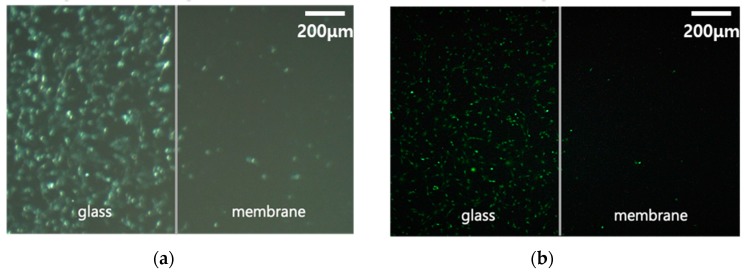
The results obtained from the anti-adhesion test in this study. (**a**) bright-field and (**b**) confocal microscopy.

To evaluate the anti-adhesion efficacy and cell test results for the alginate/CMC/PEO nanofiber film in vitro, cell experiments using NIH/3T3 were performed. Basically, formulations inserted into the human body should not be toxic, so the cell cytotoxicity of the alginate/CMC/PEO films was characterized by SEM and AFM. The cytotoxicity results were confirmed using the WST-8 Assay Kit, and no significant toxicity was found for any of the electrospun films prepared under the conditions listed in Table 1 (Figure 5). To confirm the anti-adhesion effects, adhesion tests were performed under the conditions shown in Scheme 2 with LIVE/DEAD assays. The cell adhesion evaluation for the 9% alginate/CMC/PEO nanofiber film showed better attachment to the glass than the film (Figure 6). Generally, cells are known to attach poorly to glass surfaces. The results of this study suggest that we can expect to decrease physical adhesion by applying electrospun films with low cell attachment.

### 3.4. Evaluation of Lidocaine Release Behavior by Crosslinking Degree of Nanofibrous Films

**Figure 7:**
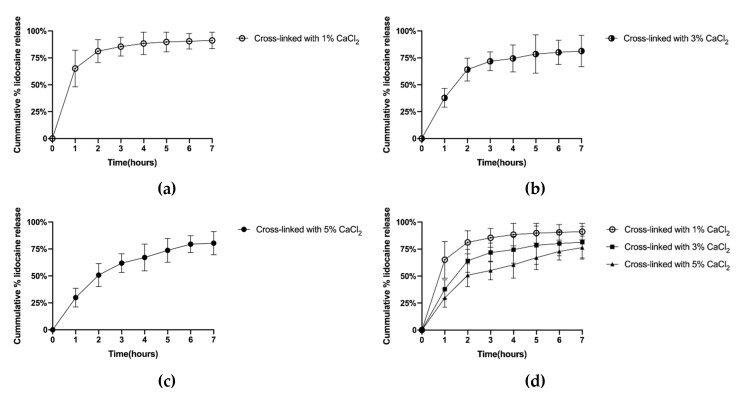
Evaluation of the lidocaine release behavior of 9% (*w/v*) alginate/CMC/PEO nanofiber films by crosslinking degree. All data were obtained from HPLC. (**a**) is the release behavior of the crosslinked alginate/CMC/PEO nanofiber film with 1% (*w/v*) CaCl_2_. (**b**,**c**) show the release behavior of the crosslinked alginate/CMC/PEO nanofiber films with 3% and 5% (*w/v*) CaCl_2_. (**d**) is a comparative analysis of the HPLC data in (**a**–**c**).

Most drugs have a recommended amount, and using an amount above or below the recommended amount can lead to poor results. Therefore, many studies are performed to introduce various functions through formulation development. In this study, the drug release behavior of an electrospun film containing lidocaine was evaluated. Lidocaine is a widely used anesthetic and is considered safe for use in the medical field. Often, drugs are misused, and abuse is harmful to health. Therefore, it is very important to develop a formulation with stable drug release behavior. In this study, the degree of crosslinking of the alginate/CMC/PEO nanofiber film was controlled to stabilize the drug release behavior and to regulate the amount of drug release (Figure 7). The stable release of lidocaine was confirmed by HPLC. The release was observed for 7 h in total, and the results confirmed that rapid release occurred under for an alginate/CMC/PEO film crosslinked with 1% (*w/v*) CaCl_2_ (Figure 7). The lidocaine release amount was nearly 70% at 1 h, which was higher than that of the alginate/CMC/PEO films crosslinked with 3% and 5% (*w/v*). Thus, the higher the degree of crosslinking, the less lidocaine released per hour from the alginate/CMC/PEO film (Figure 7). The maximum time for the stable drug release is expected to be longer for films crosslinked with 5% CaCl_2_. However, as an anti-adhesion barrier, a film should be able to release drugs over a long period and remain in place for a long period when inserted into the human body to prevent tissue adhesion. Future work is necessary to develop a system that can release drugs stably over a long period of time.

## 4. Conclusions

In this study, we prepared lidocaine-loaded alginate/CMC/PEO nanofiber films by electrospinning. In previous studies, lidocaine has been found to prevent tissue adhesion after open surgery. Therefore, we developed an effective anti-adhesive barrier by controlling the degree of crosslinking to regulate lidocaine release and maximize the negative charge characteristics of alginate to reduce cell adhesion. The lidocaine-loaded alginate/CMC/PEO nanofiber film was characterized by atomic force microscopy and was made of fibers with nanoscale diameters. Furthermore, NIH/3T3 cells were evaluated for cytotoxicity and proliferation, and the results were not significantly different from those in the control group. The HPLC results showed that the lidocaine release behavior can be controlled by the crosslinking degree of the alginate/CMC/PEO nanofiber film, and the higher the crosslinking degree, the lower the release rate of lidocaine per hour. In addition, the control of the maximum drug release behavior time was observed to increase as the degree of crosslinking increased. Based on these results, this study shows that the drug release behavior can be controlled by using CaCl_2_ as a nontoxic crosslinking agent to produce a good anti-adhesion barrier that can prevent unnecessary tissue adhesion at a surgical site.

## Figures and Tables

**Table 1:** Formulation ratios used in this study.

Final Concentration (in 10 mL of 2% Lidocaine Solution)	Formulation		
	Sodium Alginate	CMC ^1^	PEO ^2^
5% (*w/v*)	225 mg	225 mg	50 mg
7% (*w/v*)	315 mg	315 mg	70 mg
9% (*w/v*)	405 mg	405 mg	90 mg

^1^ CMC = carboxymethyl cellulose; ^2^ PEO = polyethylene oxide.

**Table 2:** Various concentrations of crosslinking agent used in this study.

Concentration of CaCl_2_ (in 10 mL of 95% Ethanol)		
1% (*w/v*)	3% (*w/v*)	5% (*w/v*)

**Table 3:** Conditions of lidocaine-loaded alginate/CMC/PEO nanofiber films to confirm the lidocaine release test.

Electrospun Film Condition	Crosslinked with CaCl_2_ Concentration
9% (*w/v*) alginate/CMC/PEO nanofiber film	1% (*w/v*) CaCl_2_
	3% (*w/v*) CaCl_2_
	5% (*w/v*) CaCl_2_
